# Tedizolid Versus Linezolid for the Treatment of Acute Bacterial Skin and Skin Structure Infection: A Systematic Review and Meta-Analysis

**DOI:** 10.3390/antibiotics8030137

**Published:** 2019-09-04

**Authors:** Shao-Huan Lan, Wei-Ting Lin, Shen-Peng Chang, Li-Chin Lu, Chien-Ming Chao, Chih-Cheng Lai, Jui-Hsiang Wang

**Affiliations:** 1 School of Pharmaceutical Sciences and Medical Technology, Putian University, Putian 351100, China; 2 Department of Orthopedic, Chi Mei Medical Center, Tainan 71004, Taiwan; 3 Department of Physical Therapy, Shu Zen Junior College of Medicine and Management, Kaohsiung 82144, Taiwan; 4 Yijia Pharmacy, Tainan 70846, Taiwan; 5 School of Management, Putian University, Putian 351100, China; 6 Department of Intensive Care Medicine, Chi Mei Medical Center, Liouying 73657, Taiwan; 7 Department of Internal Medicine, Kaohsiung Veterans General Hospital, Tainan Branch, Tainan 71051, Taiwan; 8 Department of Internal Medicine, Division of Infection Disease, Kaohsiung Veterans General Hospital, Tainan Branch, Tainan 71051, Taiwan

**Keywords:** tedizolid, linezolid, acute bacterial skin and skin structure infection

## Abstract

This meta-analysis aims to assess the efficacy and safety of tedizolid, compared to linezolid, in the treatment of acute bacterial skin and skin structure infection (ABSSSI). PubMed, Web of Science, EBSCO (Elton B. Stephens Co.), Cochrane Library, Ovid Medline and Embase databases were accessed until 18 July 2019. Only randomized controlled trials (RCTs) comparing the efficacy of tedizolid with linezolid for adult patients with ABSSSIs were included. The outcomes included the clinical response, microbiological response, and risk of adverse events (AEs). A total of four RCTs involving 2056 adult patients with ABSSSI were enrolled. The early clinical response rate was 79.6% and 80.5% for patients receiving tedizolid and linezolid, respectively. The pooled analysis showed that tedizolid had a non-inferior early clinical response rate to linezolid (odds ratio (OR) = 0.96, 95% confidence interval (CI) = 0.77–1.19, *I*^2^ = 0%). The early response rate was similar between tedizolid and linezolid among patients with cellulitis/erysipelas (75.1% vs. 77.1%; OR = 0.90, 95% CI = 0.64–1.27, *I*^2^ = 25%), major cutaneous abscess (85.1% vs. 86.8%; OR = 0.93, 95% CI = 0.42–2.03, *I*^2^ = 37%) and wound infection (85.9% vs. 82.6%; OR = 1.29, 95% CI = 0.66–2.51, *I*^2^ = 45%). For methicillin-resistant *Staphylococcus aureus* patients, tedizolid had a favorable microbiological response rate of 95.2% which was comparable to linezolid (94%) (OR = 1.19, 95% CI = 0.49–2.90, *I*^2^ = 0%). In addition to the similar risk of treatment-emergent AEs (a serious event, the discontinuation of the study drug due to AEs and mortality between tedizolid and linezolid), tedizolid was associated with a lower risk of nausea, vomiting and abnormal neutrophil count than linezolid. In conclusion, once-daily tedizolid (200 mg for six days) compared to linezolid (600 mg twice-daily for 10 days) was non-inferior in efficacy in the treatment of ABSSSI. Besides, tedizolid was generally as well tolerated as linezolid, and had a lower incidence of gastrointestinal AEs and bone marrow suppression than linezolid.

## 1. Introduction

Acute bacterial skin and skin structure infections (ABSSSIs) including cellulitis, erysipelas, major cutaneous abscess, and wound infection are one of the most frequent illnesses requiring hospitalization for treatment [1,2,3]. Because ABSSSI can be serious infections, prompt diagnosis and the use of an appropriate antibiotic should be key to management. Gram-positive bacterial *Streptococcus* spp. and *Staphylococcus* spp. are the most common reported pathogens in this clinical entity. In this era of antibiotic resistance, the prevalence of antibiotic-resistant bacteria, particularly methicillin-resistant *Staphylococcus aureus* (MRSA) in ABSSSI is increasing and their presence has led to significant increases in morbidity, mortality and overall healthcare costs [4,5]. In order to improve the outcome of patients with MRSA-associated ABSSSI, the identification of populations at risk and early use of effective antibiotics are essential. Currently, vancomycin, teicoplanin, daptomycin, linezolid, and ceftaroline are the recommended antibiotics for ABSSSI due to MRSA [6,7]. Recently, a number of new antimicrobials with enhanced activity against MRSA have been developed and approved for the treatment of ABSSSI. Tedizolid—a novel oxazolidinone—represents one of the treatment options with favorable pharmacokinetic characteristics and a good safety profile [8]. However, the efficacy and safety of these new drugs are only reported in limited studies [9,10,11,12,13], and the experience in real-world clinical practice remains scarce. Moreover, it remains unclear whether the clinical efficacy and the risk of adverse events (AEs) of tedizolid is not inferior to those of the first oxazolidinone—linezolid or not. Therefore, we conduct this meta-analysis to compare these two oxazolidinones (tedizolid and linezolid) in the treatment of ABSSSI.

## 2. Methods

### 2.1. Study Search and Selection

All randomized controlled trials (RCTs) were identified by a systematic review of databases including PubMed, Web of Science, EBSCO (Elton B. Stephens Co.), Cochrane Library, Ovid Medline and Embase until 18 July 2019 using the following terms: “tedizolid”, “torezolid”, “sivextro”, and “acute bacterial skin and skin structure infection”. Only RCTs that compared the clinical efficacy and the risk of AEs of tedizolid and linezolid for the treatment of adult patients (≥18 years) with ABSSSI were included. The exclusion criteria were: (1) case reports or case series; (2) single arm studies; (3) pediatric studies; (4) conference abstract; (5) pharmacokinetic study; and (6) in vitro study. To avoid bias, two authors (Chang and Lan) were responsible for searching for and examining the articles independently. Regarding disagreements, another author (Lai) would help to resolve the issue and make the final decision. The data included authors, year of publication, study design and duration, the demographic characteristics of patients, type of infections, the clinical response and the risk of AEs. The Cochrane Risk for Bias Assessment tool [14] was used to assess the risk of bias for RCTs in this meta-analysis.

### 2.2. Definition and Outcome

The intention-to-treat (ITT) population was defined as all randomized patients and the clinically evaluable (CE) population who received tedizolid or linezolid according to the study protocol and had a complete assessment of clinical response. The microbiological evaluable (ME) population included the CE population who had a confirmed pathogen at baseline. The primary outcome was an early clinical response at 48–72 h. Secondary outcomes included the clinical response at the end of treatment (EOT), post-therapy evaluation (PTE) (7–14 days after EOT), and the risk of AEs, including treatment-emergent AEs (TEAEs), serious AEs, discontinuation because of AEs, and mortality. 

### 2.3. Statistical Analysis

Statistical analyses were conduct using the software Review Manager, version 5.3. The degree of heterogeneity was evaluated with the *Q* statistic generated from the χ^2^ test. The proportion of statistical heterogeneity was assessed by the *I*^2^ measure. Heterogeneity was defined as significant when the *p*-value was less than 0.10 or *I*^2^ was more than 50%. The fixed-effect model and the random-effects model were applied when the data was considered as homogenous and heterogeneous, respectively. Pooled odds ratio (OR) and 95% confidence intervals (CIs) were calculated and a *p*-value <0.05 was considered as statistical significance. Sensitivity analyses were conducted by excluding or subgrouping studies to reduce potential confounding effects.

## 3. Results

### 3.1. Study Selection and Characteristics

**Figure 1:**
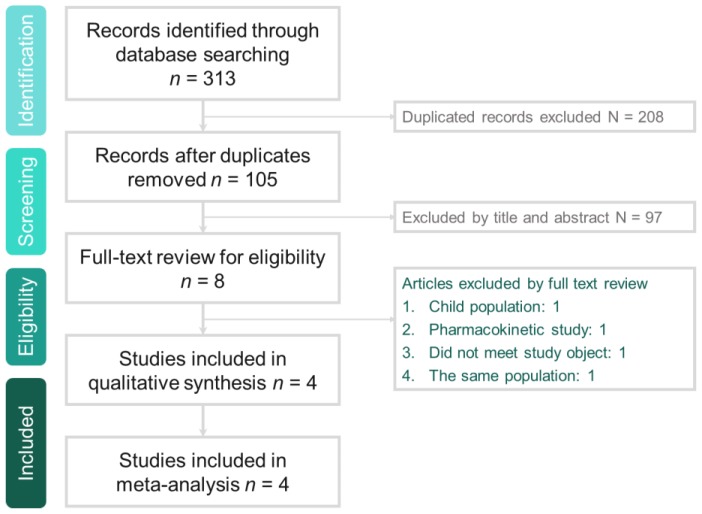
The algorithm of study selection.

**Figure 2:**
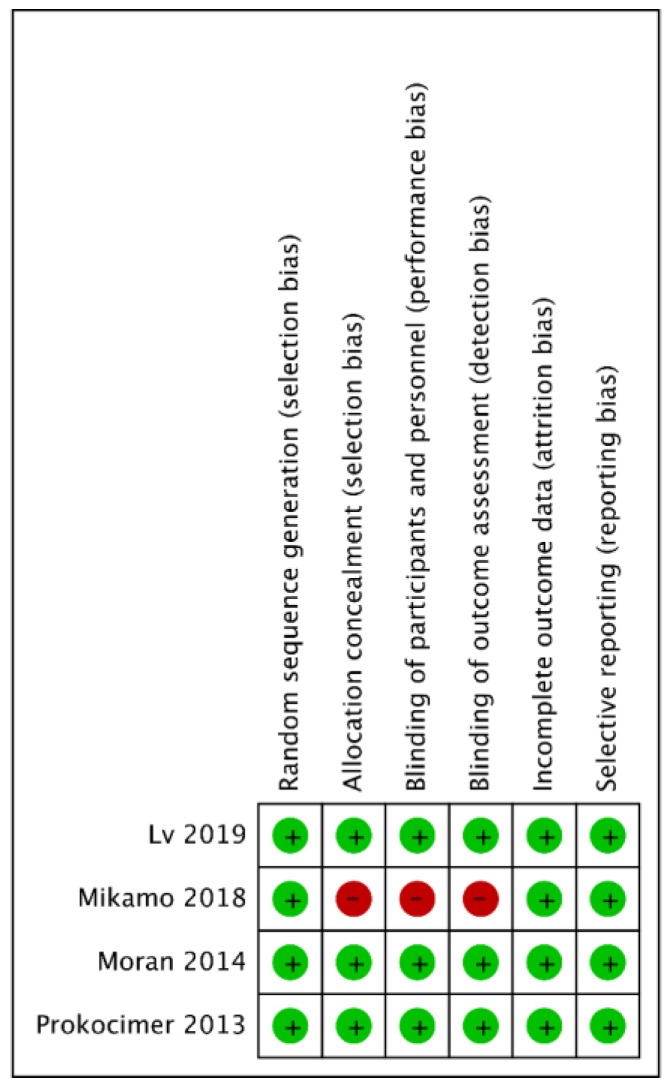
The risk of bias in each domain.

The search program generated 313 reference; 105 articles were screened after excluding 208 duplicated articles. Finally, eight articles were identified for full-text review for eligibility and only four studies [9,10,11,13] designed to compare the clinical efficacy and safety of tedizolid and linezolid in the treatment of patients with ABSSSI were enrolled in this meta-analysis (Figure 1 and Appendix A). Overall, this meta-analysis included a total of 2056 patients (1048 in the tedizolid group and 1008 in the linezolid group). Two studies [11,13] were primarily conducted in western countries, one [9] was primarily in Asian countries and one [10] was only in Japan (Table 1). Except for the Prokocimer et al. study [13], which compared only the oral form of tedizolid and linezolid, the other three studies compared an initial intravenous injection followed by oral use. In addition, the treatment duration of tedizolid and linezolid was different (6 days vs. 10 days) (Table 1). The demographic characteristics of the patients is listed in Table 2. The mean age of the enrolled patients in the Mikamo et al. study [10] was older than those of the other studies [9,11,13]. Overall, males comprised about 60% of the patients, but only less than 5% of patients had secondary bacteremia due to ABSSSI. Finally, cellulitis/erysipelas was the most common type of infection, comprising more than 50% of patients, followed by wound infection (≅27%) and major cutaneous abscess (≅20%) (Table 3). The risk of bias in each study is shown in Figure 2. Only one study [10] exhibited a high risk of bias in the domains of allocation concealment, performance and detection bias.

### 3.2. Clinical Efficacy

**Figure 3:**
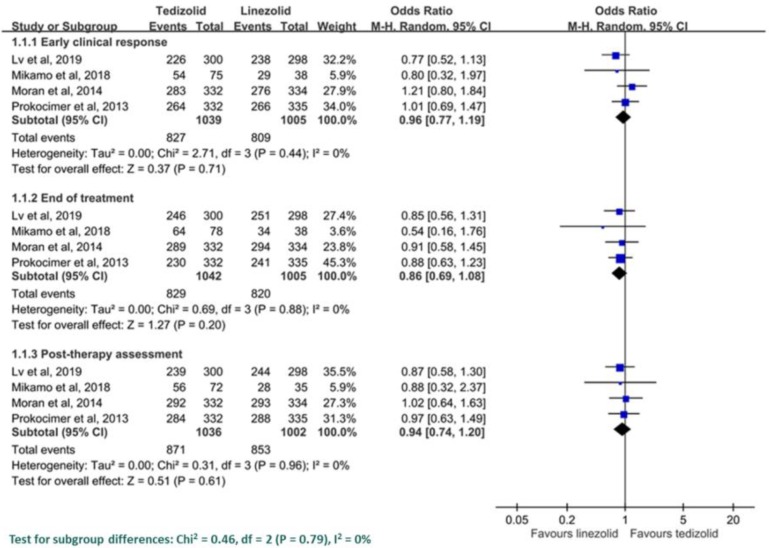
The clinical response between tedizolid and linezolid.

**Figure 4:**
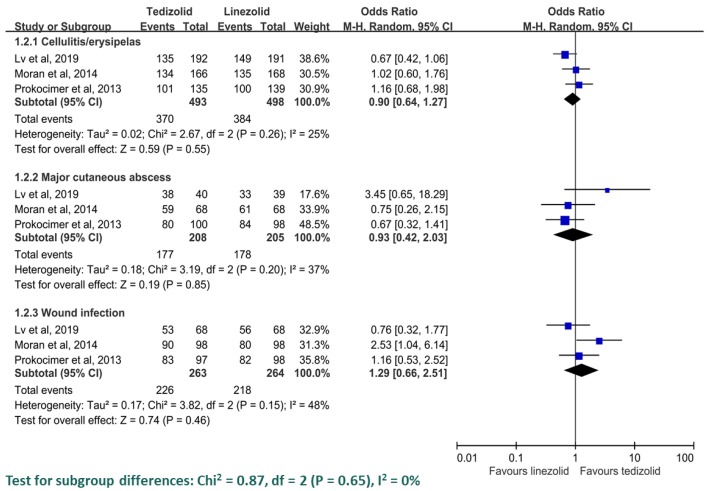
The early clinical response in each type of infection between tedizolid and linezolid.

Among all four trials, the early clinical response rate was 79.6% (827/1039) and 80.5% (809/1005) in the ITT group of patients who received tedizolid and linezolid, respectively. Pooled analysis showed that tedizolid had a similar early clinical response rate as linezolid (OR = 0.96; 95% CI = 0.77–1.19, *I*^2^ = 0%, Figure 3). Sensitivity analysis after deleting an individual study each time showed the same findings. Furthermore, the clinical response rates of tedizolid and linezolid were similar at EOT (79.6% vs. 81.2%; OR = 0.86, 95% CI = 0.69–1.08, *I*^2^ = 0%) and PTE (84.1% vs. 85.1%; OR = 0.94, 95% CI = 0.74–1.20, *I*^2^ = 0%) among the ITT population. The pooled analysis of three studies [9,11,13] reported the clinical response rate at EOT and PTE in the CE population, and no difference was observed at EOT (86.0% vs. 89.0%; OR = 0.79, 95% CI, 0.59–1.67, *I*^2^ = 0%), and PTE (93.5% vs. 95.0%; OR = 0.64, 95% CI = 0.38–1.09, *I*^2^ = 0%). In the subgroup analysis according to infection type, the pooled analysis of three RCTs [9,11,13] showed the early response rate was similar between tedizolid and linezolid among patients with cellulitis/erysipelas (75.1% vs. 77.1%; OR = 0.90, 95% CI = 0.64–1.27, *I*^2^ = 25%), major cutaneous abscess (85.1% vs. 86.8%; OR = 0.93, 95% CI = 0.42–2.03, *I*^2^ = 37%) and wound infection (85.9% vs. 82.6%; OR = 1.29, 95% CI = 0.66–2.51, *I*^2^ = 45%) (Figure 4).

### 3.3. Minimum Inhibitory Concentration (MIC) and Microbiological Response among the MRSA Population

All four studies [9,10,11,13] reported the minimum inhibitory concentrations (MICs) for MRSA isolates for tedizolid and linezolid which were ≥0.5 mg/L and ≤2–4 mg/L, respectively. MIC_90_ of tedizolid against MRSA was 0.25 mg/L in one study [13] and 0.5 mg/L in the other two studies [10,11]. MIC_90_ of linezolid against MRSA was 2 mg/L in three studies [10,11,13]. Three RCTs [9,10,11] reported the microbiological response among the microbiologically evaluable (ME) MRSA population and the subgroup analysis revelated that tedizolid had a favorable microbiological response rate of 95.2%, which was comparable to linezolid (94%) (OR = 1.19, 95% CI = 0.49–2.90, *I*^2^ = 0%).

### 3.4. Adverse Events

**Figure 5:**
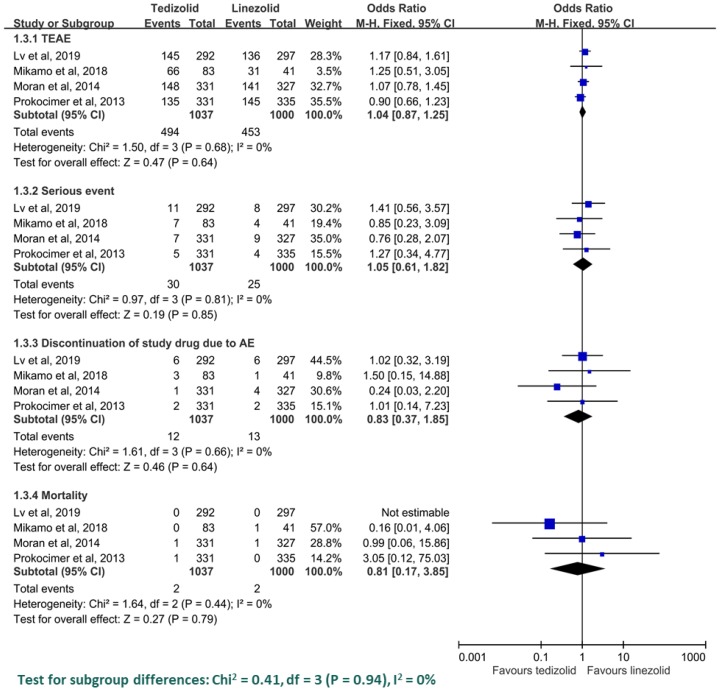
The risk of an adverse event with tedizolid and linezolid.

In the pooled analysis of the risk of AE, tedizolid was associated with a similar risk as linezolid in terms of TEAEs (OR = 1.04, 95% CI = 0.87–1.25, *I*^2^ = 0%), serious events (OR = 1.05, 95% CI = 0.61–1.82, *I*^2^ = 0%), discontinuation of study drug due to AEs (OR = 0.83, 95% CI = 0.37–1.8, *I*^2^ = 0%) and mortality (OR = 0.81, 95% CI = 0.17–3.85, *I*^2^ = 0%) (Figure 5). The most common AE among patients receiving tedizolid was nausea (6.8%), followed by diarrhea (3.3%) and vomiting (2.6%). Among these gastrointestinal AEs, the risk of nausea and vomiting was lower in the tedizolid group than linezolid (nausea: OR = 0.68, 95% CI = 0.49–0.94, *I*^2^ = 0%; vomiting: OR = 0.56, 95% CI = 0.34–0.96, *I*^2^ = 0%), but no difference was observed in terms of diarrhea (OR = 0.74, 95% CI = 0.47–1.18, *I*^2^ = 0%). Regarding the risk of bone marrow suppression, tedizolid was associated with a lower rate of abnormal neutrophil count than linezolid (1.3% vs. 3.9%; OR = 0.36, 95% CI = 0.17–0.76, *I*^2^ = 0%). Regarding the risk of abnormal platelet count, tedizolid was also associated a lower risk than linezolid (4.2% vs. 6.8%; OR = 0.61, 95% CI = 0.25–1.49, *I*^2^ = 0%), but the difference did not reach statistical significance.

## 4. Discussion

This meta-analysis included four RCTs enrolling 2056 patients (*n* = 1048 tedizolid and *n* = 1008 linezolid) with ABSSSIs. Overall, our findings indicated that tedizolid was non-inferior to linezolid in the treatment of ABSSSI, and these findings were supported by the following analysis. First, the early clinical response rate of tedizolid was 79.6% among the ITT population, which was comparable to linezolid (80.5%). This non-inferiority between tedizolid and linezolid remained the same in the sensitivity analysis. This finding was consistent with previous post-hoc analyses [15,16,17] of two phase 3 studies in which the non-inferiority of tedizolid to linezolid in terms of early responses remained consistent across various populations, including body mass index ≥30 kg/m^2^, diabetes, intravenous drug users, elderly patients of age ≥65 years, patients with renal impairment and different disease severity/sites. Second, the clinical responses at test and PTE among the ITT and CE population were similar between the tedizolid and linezolid groups. Third, as in the previous report [15], no difference in the various types of infection—cellulitis/erysipelas, major cutaneous abscess, and wound infection—was observed in terms of early clinical response in the comparison between tedizolid and linezolid. In summary, the clinical efficacy of six-day tedizolid in the treatment of ABSSSI was non-inferior to 10-day linezolid based on this meta-analysis.

In addition to clinical efficacy, the microbiological response of the antibiotics in the treatment of acute bacterial infections should be another important concern. In the treatment of ABSSSI, MRSA is a serious concern, which is a common pathogen and remains resistant to many common antibiotics. Among these enrolled RCTs, all MICs of tedizolid against MRSA were ≤0.5 mg/L, and the MIC_90_ of tedizolid was only 0.5 mg/L, which was lower than those of linezolid (2–4 mg/L). The similar excellent in vitro activity of tedizolid has been demonstrated in many previous studies [18,19,20,21]. A pooled analysis [18] of eighteen studies showed that the overall in vitro activity of tedizolid against 10,119 MRSA isolates was estimated to be 0.25 mg/L and 0.5 mg/L for MIC_50_ and MIC_90_, respectively, and the pooled MRSA susceptibility of tedizolid was estimated at 99.6% (95% CI = 99.5–99.8%). Besides the findings of these in vitro studies, our meta-analysis found that tedizolid exhibited a favorable microbiological response of 95.2% among the ME-MRSA population, which was non-inferior to linezolid. In the previous analysis [15], the early clinical response and clinical response at PTE of tedizolid for the treatment of MRSA was 80.9% (114/141) and 84.8% (151/175), respectively, and both of these response rates were similar to those of linezolid. Therefore, even for MRSA-associated ABSSSI, the clinical and microbiological response of tedizolid was non-inferior to linezolid, and these findings suggest that tedizolid could be a promising alternative for the treatment of MRSA-associated ABSSSI.

Finally, clinicians should take safety issues into consideration while prescribing antibiotics to treat acute bacterial infections. Initially in the pooled analysis of the risk of AE, tedizolid was associated with a similar risk to linezolid in terms of TEAE, serious events, discontinuation of the study drug due to AE and mortality. Specifically, the most common AE of tedizolid was gastrointestinal discomforts, including nausea and vomiting, but the risks of nausea and vomiting were lower in the tedizolid group than the linezolid group. Moreover, the risk of bone marrow suppression was also lower in the tedizolid group than the linezolid group. Overall, our findings suggest that tedizolid was at least as tolerable as linezolid.

This meta-analysis has two major strengths. First, it is updated and includes two more studies than the previous analysis [15]. Second, it included many Asian patients, which generalize the findings better than previous reports. However, several limitations of this meta-analysis should be noted. First, although a short course of tedizolid may be associated with less hospital cost than a 10-day course of linezolid, the cost-effectiveness was not evaluated in this analysis. Second, other outcomes, such as recurrence and relapse after a short course of tedizolid treatment, were not assessed in this analysis. Further study is warranted to clarify these two issues.

## 5. Conclusions

This meta-analysis suggests that once-daily tedizolid for six days exhibits non-inferior efficacy compared with twice-daily linezolid for 10 days in the treatment of ABSSSI. In addition, tedizolid was generally as well tolerated as linezolid, and even had a lower incidence of gastrointestinal AEs and bone marrow suppression compared to linezolid. 

## Figures and Tables

**Table 1:** Characteristics of included studies. MRSA = methicillin-resistant *Staphylococcus aureus.*

The Study, Published Year	Study Design	Study Period	Number of Patients		Dose Regimen	
			Tedizolid	Linezolid	Tedizolid	Linezolid
Prokocimer et al., 2013 [13]	Randomized, double-blind, multicenter, multinational, non-inferiority trial	2010–2011	332	335	oral 200 mg daily × 6 days	oral 600 mg twice daily × 10 days
Moran et al., 2014 [11]	Randomized, double-blind, multinational, non-inferiority trial	2011–2013	332	334	intravenous 200 mg daily × 6 days with optional step-down	intravenous 600 mg twice daily × 10 days with optional step down
Mikamo et al., 2018 [10]	Prospective, randomized, open-label, multicenter trial	2013–2016	84	41	intravenous /oral 200 mg daily × 6 days	intravenous /oral 600 mg twice daily × 10 days
Lv et al., 2019 [9]	Randomized, double-blind, multicenter, non-inferiority trial	2014–2016	300	298	intravenous /oral 200 mg daily × 6 days	intravenous /oral 600 mg twice daily × 10 days

**Table 2:** Demographic features of enrolled cases.

Study, Year	Age		Male Sex, Number (%)		Bacteremia, Number (%)		Number with MRSA at Baseline	
	Tedizolid	Linezolid	Tedizolid	Linezolid	Tedizolid	Linezolid	Tedizolid	Linezolid
Prokocimer et al., 2013 [13]	43.6 (14.96)	43.1 (15.06)	204 (61.4)	198 (59.1)	NA	NA	88	90
Moran et al., 2014 [11]	46 (17–86)	46 (15–89)	225 (68)	214 (64)	7 (2)	12 (4)	53	56
Mikamo et al., 2018 [10]	63.4 (16.5)	63.3 (16.2)	55 (65.5)	28 (68.3)	4 (4.8)	2 (4.9)	32	13
Lv et al., 2019 [9]	45.7 (18–85)	47.5 (18–85)	209 (69.7)	192 (64.4)	5 (1.7)	3 (1.0)	29	32

**Table 3:** Type of infection.

Study	Cellulitis/Erysipelas, Number (%)		Major Cutaneous Abscess, Number (%)		Wound, Number (%)	
	Tedizolid	Linezolid	Tedizolid	Linezolid	Tedizolid	Linezolid
Prokocimer et al., 2013 [13]	135 (40.4)	139 (41.5)	100 (30.1)	98 (29.3)	97 (29.2)	98 (29.3)
Moran et al., 2014 [11]	166 (50)	168 (50)	68 (20)	68 (20)	98 (30)	98 (29)
Mikamo et al., 2018 [10]	44 (52.4)	22 (53.7)	3 (3.6)	2 (4.9)	16 (19.0)	10 (24.4)
Lv et al., 2019 [9]	192 (64.0)	191 (64.1)	40 (13.3)	39 (13.1)	68 (22.7)	68 (22.8)

## Appendix A. Search Strategy

**PubMed search strategy—last searched on 18 July 2019**		**Results**
1	Search Search ((Sivextro[Title/Abstract]) OR Tedizolid[Title/Abstract]) OR Torezolid[Title/Abstract]	269
2	Search ((Acute Bacterial Skin[Title/Abstract] AND Skin Structure Infection[Title/Abstract])) OR ABSSSI[Title/Abstract]	178
3	1 AND 2	
4	Search ((((“acute bacterial skin[Title/Abstract] AND skin structure infections”[Title/Abstract])) OR ABSSSI[Title/Abstract])) AND (((Sivextro[Title/Abstract]) OR Tedizolid[Title/Abstract]) OR Torezolid[Title/Abstract])	34
**Web of Science search strategy—last searched on 18 July 2019**		**Results**
1	Topic: (Sivextro) OR Topic: (Torezolid) OR Topic: (Tedizolid)	277
2	Topic: (“acute bacterial skin and skin structure infections”) OR Topic: (ABSSSI)	321
3	1 AND 2	
4	#1 AND #2	60
**EBSCO search strategy—last searched on 18 July 2019**		**Results**
1	AB Sivextro OR AB Torezolid OR AB Tedizolid	95
2	AB (acute bacterial skin and skin structure infections) OR AB ABSSSI	136
3	1 AND 2	
4	S1 AND S2	24
**Cochrane Library search strategy—last searched on 18 July 2019**		**Results**
1	(Sivextro): ti,ab,kw OR (Torezolid):ti,ab,kw OR (Tedizolid): ti,ab,kw	59
2	(acute bacterial skin and skin structure infections): ti,ab,kw OR (ABSSSI): ti,ab,kw	160
3	1 AND 2	
4	#1 AND #2	27
**Ovid Medline search strategy—last searched on 18 July 2019**		**Results**
1	(Sivextro or Torezolid or Tedizolid).ab	324
2	((Acute bacterial skin and skin structure infections) or ABSSSI).ab.	433
3	1 AND 2	
4	1 and 2	74
**Embase search strategy—last searched on 18 July 2019**		**Results**
1	Sivextro: ti,ab,kw OR torezolid: ti,ab,kw OR tedizolid: ti,ab,kw	362
2	‘acute bacterial skin’: ti,ab,kw AND ‘skin structure infections’: ti,ab,kw OR absssi: ti,ab,kw	534
3	1 AND 2	
4	#1 AND #2	93
